# Endocrine toxicities in immune checkpoint inhibitors and tyrosine kinase inhibitors combined treatment: from clinical trials to real-life practice

**DOI:** 10.1007/s12020-026-04635-2

**Published:** 2026-05-02

**Authors:** Sabrina Chiloiro, Maria Grazia Maratta, Simone Antonio De Sanctis, Flavia Costanza, Ernesto Rossi, Emanuele Vita, Antonella Giampietro, Francesco Pavese, Olga Martelli, Ida Paris, Antonio Bianchi, Giovanni Schinzari, Laura De Marinis, Anna Fagotti, Giampaolo Tortora, Alfredo Pontecorvi

**Affiliations:** 1 School of Medicine and Surgery, Università Cattolica del Sacro Cuore, Rome, Italy; 2 Department of Medicine, Endocrinology and Diabetology, Fondazione Policlinico Universitario A. Gemelli IRCCS, Largo A. Gemelli, Rome, Italy; 3 Comprehensive Cancer Center, Dipartimento Scienze della Salute della Donna, del Bambino e di Sanità Pubblica, Fondazione Policlinico Universitario Agostino Gemelli, IRCCS, Rome, Italy; 4 Dipartimento Scienze della Salute della Donna, del Bambino e di Sanità Pubblica, Fondazione Policlinico Universitario Agostino Gemelli, IRCCS, Rome, Italy; 5 Dipartimento Scienze della Vita e Sanità Pubblica, Università Cattolica del Sacro Cuore, Rome, Italy

**Keywords:** Immune checkpoint inhibitors, Tyrosine kinase inhibitors, Combination therapy, Side effects, Adverse events, Cancer treatment

## Abstract

Combination therapies with immune checkpoint inhibitors (ICIs) and tyrosine kinase inhibitors (TKIs) have revolutionized the landscape of cancer treatment, improving the quality of life and overall survival of patients. A deep knowledge of the side effects of ICIs and TKIs combination therapy is mandatory to ensure patient compliance and improve clinical outcomes. Both ICIs and TKIs may cause endocrinopathies such as thyroid dysfunction, adrenal insufficiency, hypophysitis, and diabetes mellitus. To avoid life-threatening conditions and improve patient’s compliance and outcomes, an early diagnosis of endocrine toxicity should be achieved and a multidisciplinary approach involving both endocrinologists and oncologists may be beneficial. This review specifically examines the endocrine adverse events reported in the clinical trials of ICI plus TKI combined treatment, their underlying mechanisms, and practical management guidelines.

**Graphical Abstract:**

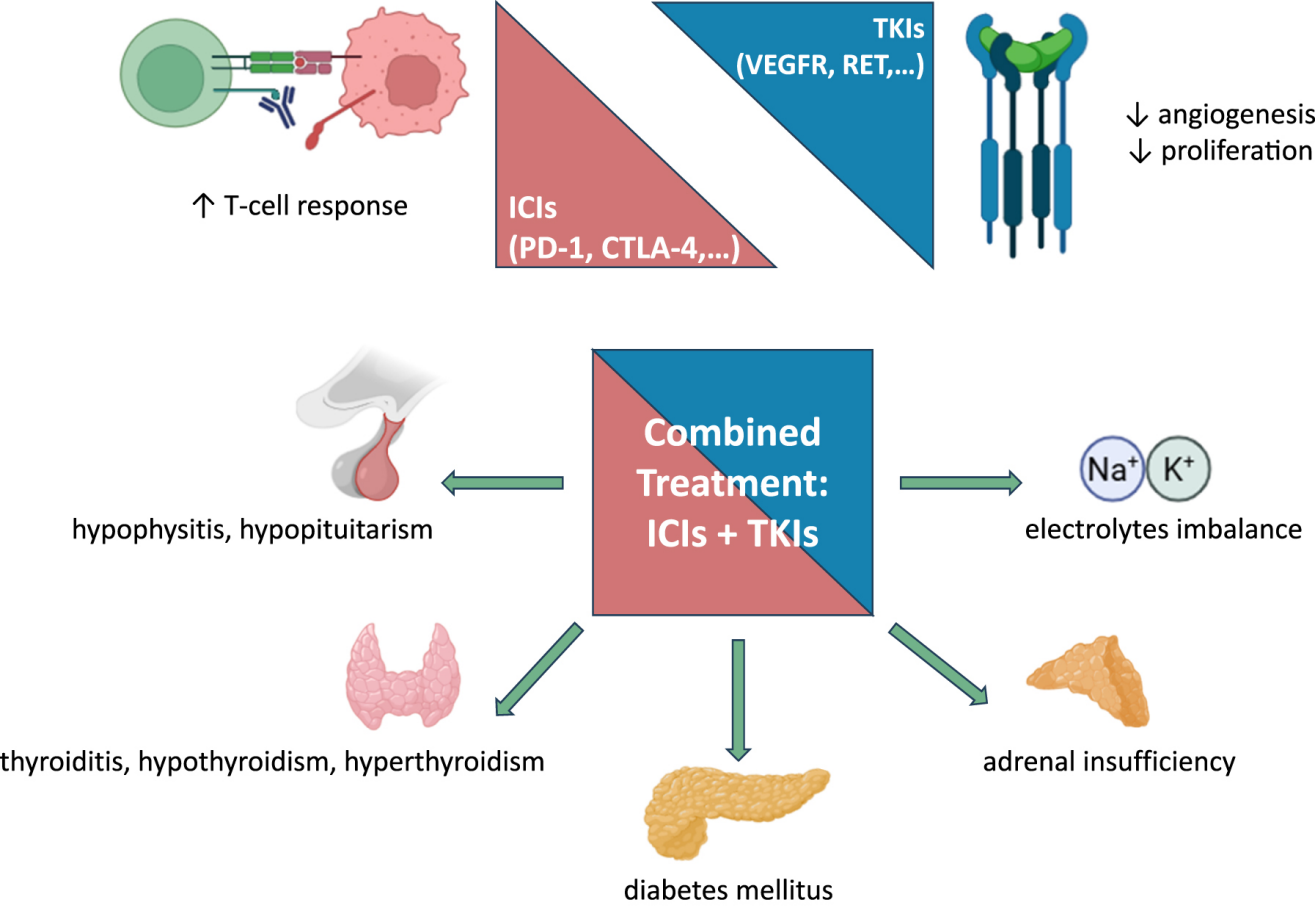

## Introduction

The combination of immune-checkpoint inhibitors (ICIs) with tyrosine kinase inhibitors (TKIs) has been recently included in the treatment of several neoplasia, revolutionizing the treatment of cancer.

The ICIs and TKIs combination therapy enables to attack the tumor by activating the immune system response against cancer cells and by blocking the neo-angiogenesis [1, 2]. TKIs act on the tumor microenvironment (TME) by blocking factors such as the vascular endothelial growth factor receptor (VEGFR), the platelet-derived growth factor receptor (PDGFR) and fibroblast growth factor receptors (FGFR), consequently reducing tumor neo-angiogenesis, neoplastic cell proliferation and neoplasm-associated immunotolerance. Meanwhile, ICIs (particularly anti-programmed death-1 -anti-PD-1- and anti-programmed death ligand-1 -anti-PD-L1 drugs) enhances the immune response against cancer antigens, promoting tumor necrosis [1].

The ICIs and TKIs combination treatment achieves a synergistic effect [2]. However, while this combination treatment greatly enhances the therapeutic efficacy, it is also associated with higher frequency of endocrine adverse events (endocrine-AEs) then monotherapies [3–6]. One of the main challenges is to distinguish between ICIs related and TKIs related endocrine-AEs, as the clinical presentations typically overlap [2, 3].

Although it is known that the development of endocrine complications is associated with a better response to immunotherapy, a potential correlation between adverse events affecting the endocrine system and clinical outcomes in patients receiving combination therapy with ICI+TKI has not yet been thoroughly investigated in the few trials reported in the literature.

However, understanding endocrine-AEs and their underlying biological mechanisms is critical to optimizing patient management and outcomes. Thus, this review aims to examine the endocrine-AEs reported in clinical trials on ICIs plus TKIs combined treatments, the underlying mechanisms and to provide practical management advice.

### Methods and search strategy

The literature search was conducted in December 2025. We searched MEDLINE (PubMed database) with the data filter 2014-2025 using the following keywords: immune checkpoint inhibitors AND tyrosine kinase inhibitors AND (combination therapy OR combination treatment OR combined therapy OR combined treatment) AND (side effects OR adverse events OR endocrine toxicities OR adrenal insufficiency OR hypoadrenalism OR hypothyroidism OR hyperthyroidism OR hypophysitis).

The articles met the following inclusion criteria: (1) written in English; (2) published between 1 January 2014, and 28 February 2025; (3) clinical trials and randomized controlled trials. Exclusion criteria included: (1) articles written in languages other than English; (2) case reports, case series, review, systematic review, meta-analysis. We selected and analyzed relevant articles, focusing on information about endocrine toxicities in ICIs-TKIs combination therapies.

Finally, we also included in the literature review significant articles relating to the mechanisms of action and toxicity of ICIs and TKIs, the incidence of the latter, and their diagnosis and clinical management.

### Epidemiology and risk factors for endocrine-adverse events

The ICIs and TKIs combination treatment is becoming increasingly common in oncology. However, this approach has a complex and significant endocrine toxicity profile. The incidence of endocrine-AEs is significantly higher with the ICI plus TKI combination (9.1%) than with ICI (7.7%) or TKI (1.2%) monotherapy [4]. The most common endocrine-AE is thyroid dysfunction (particularly primary hypothyroidism), accounting for 67.5% of cases, followed by disorders of glucose metabolism (14%). The risk of type 1 diabetes mellitus is higher with combination therapy than with TKI monotherapy (OR 26.61), but lower than with ICI monotherapy (OR 0.63). Compared to ICI monotherapy, the risk of hypoglycemia is increased during ICI plus TKI combination treatment (OR 2.77) [4]. Hypophysitis and adrenalitis are also more prevalent during ICI and TKI combination then in monotherapy. Polyendocrine syndrome is highly incident (7.9%), but mortality rates do not differ between patients with involvement of a single gland or multiple glands [4].

Risk factors for endocrine-AEs were the female gender (particularly for thyroid dysfunction) [4–6], the older age (particular for polyendocrine syndrome) and Asian ethnicity [4, 7]. ICIs and TKIs related thyroid dysfunctions are typically associated with a previous history of thyroid disease, presence of thyroid autoantibodies, and elevated thyroid-stimulating hormone (TSH) levels [8]. A higher body mass index (BMI) is also associated with an increased risk of endocrine-AEs. Furthermore, some studies have shown that patients with melanoma or metastatic renal cell carcinoma (mRCC) on ICI plus TKI combination therapy are at a higher risk for polyglandular syndrome [4, 8–10]. Finally, patients who have already developed thyroid dysfunction with TKI monotherapy are at a higher risk of recurrence or worsening with the addition of ICIs [4].

### Mechanisms of endocrine side effects induced by the combination therapy ICI plus TKI

**Fig. 1:**
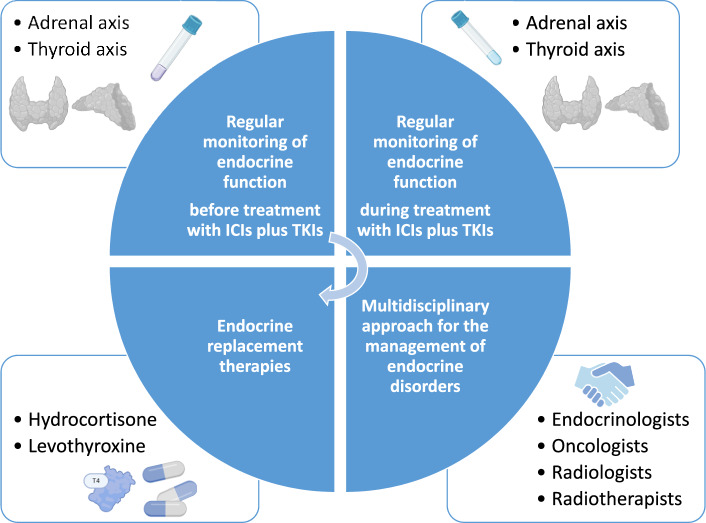
Mechanisms of endocrine toxicities of ICIs and TKIs

The etiology of endocrine-AEs related to ICI plus TKI combination treatment is multifactorial, deriving from a complex interplay between immune system activation and neo-angiogenesis [2]. ICIs are responsible for immune-related adverse events (iREs), acting through an immune-mediated destruction process against endocrine glands [2, 4, 11]. The mechanisms of endocrine toxicities of ICIs and TKIs are summarized in Fig. 1. ICIs can trigger an autoimmune response in endocrine glands, with infiltration of inflammatory cells, leading to pathological conditions such as thyroiditis, adrenalitis and hypophysitis [12–14]. Whereas TKIs affect the blood supply of endocrine glands, leading to ischemia and oxidative stress, which further impairs the function of the gland and the secretion of hormones [1].

It is known that the thyroid gland is the most affected gland of all [1, 2]. ICIs cause destructive autoimmune thyroiditis through hyperactivation of T lymphocytes, with a prevalence of cytotoxic CD8+ cells, at the systemic and intraglandular levels; there is also an increase in NK (Natural Killer) cells and CD14+CD16+ monocytes [2-4-11]. TKIs cause vascular destructive thyroiditis, with ischaemia and tissue damage. Furthermore, some studies show that other mechanisms may contribute to TKI-induced damage: for example, these drugs may cause reduced iodine uptake in the gland, possible interference with the sodium-iodine symporter, and interference with the transport and peripheral metabolism of hormones through modulation of the activity of the deiodinase enzyme [1]. Some drugs such as imatinib and dasatinib cause an increase in the expression of isoform 3 of the enzyme, resulting in the inactivation of T4 and a reduction in circulating thyroid hormones, while other drugs such as vandetanib act by reducing the conversion of T4 to T3, with the same consequence [1].

However, in patients treated with the ICI plus TKI combination therapy, the diagnostic challenge is identifying the specific agent responsible for the AE, given the overlap between immune and vascular pathogenesis in these toxicities [15–17].

### Diagnosis and treatment of endocrine toxicities induced by ICIs and TKIs

Endocrine toxicities induced by ICIs and TKIs require a prompt diagnosis and management, being potential life-threatening conditions [12, 18–24].

Clinicians must raise their awareness of the symptoms referred by patients on ICIs and TKIs combination therapy. Symptoms of endocrine-AEs are often non-specific and may overlap with other systemic AEs of cancer therapy [12]. Biochemical evaluation is crucial and should include baseline and periodic assessment throughout ICIs and TKIs combination treatment, including at least TSH, free thyroxine (fT4), morning cortisol, and adrenocorticotropic hormone (ACTH) [19].

The recommendations of the main scientific societies regarding the diagnosis and monitoring of endocrine toxicities are summarized in Table 1 and Table 2.

**Table 1:** Guidelines from the main scientific societies regarding the onset and monitoring of endocrine toxicities during therapy with ICIs

		ASCO (2018)	British Society for Endocrinology (2018)	French Endocrine Society (2019)	ESE (2022)	AIOM (2023)	ESMO (2023)
Baseline	Thyroid toxicity	TSH, fT4	TSH, fT4	TSH, fT4,	TSH, fT4	TSH, fT4	TSH, fT3, fT4
	Adrenal toxicity	Electrolytes cortisol	Electrolytes cortisol	Sodium, cortisol	Cortisol, ACTH if cortisol < 500 nmol/L	Cortisol, ACTH if cortisol < 500 nmol/L	Cortisol
	Type 1 diabetes	Glycemia	Glycemia	Glycaemia (only with anti-PD-1/ PD-L1)	Not available	Glycemia	Glycaemia and HbA1c
	Pituitary toxicity	Electrolytes TSH, fT4, cortisol	ACTH, cortisol TSH, fT4 Prolactin, testosterone/estradiol, LH/FSH	ACTH, cortisol TSH, fT4 LH, FSH, testosterone/estradiol	Electrolytes Glycaemia TSH, fT4, cortisol	Not available	TSH, fT4, cortisol
	Gonadal toxicity	Not available	testosterone/estradiol, LH/FSH	Not available	Not available	Not available	Not available
Follow-up	Thyroid toxicities	TSH, fT4 every 4-6 weeks	Not available	TSH, fT4 before each ICI dose during first 6 months of treatment every second cycle from month 6 to month 12	TSH, fT4 before every ICI dose from months 1-6; every 2–3 months from 6-12 months	TSH, fT4 every 2-3 weeks in case of alterations; every 4-6 weeks during active treatment; every 6-12 weeks after the treatment for 1 year follow-up	TSH, fT3, fT4 every dose from months 1-3 in anti-PD-(L)-1 then every second dose every dose in anti-CTLA-4 and ICI combination, every 4-8 weeks after cycle 4
	Adrenal toxicity	Electrolytes Cortisol every 4-6 weeks	Not available	Sodium, cortisol: every ICI dose from 1-6 months at every second cycle from 6-12 months.	Periodically	Periodically	If adrenal insufficiency is suspected: 8 am serum cortisol, 8 am ACTH, or LH, FSH and ACTH
	Type 1 diabetes	Glycemia every 4-6 weeks	Not available	Glycemia every ICI dose month 1-6 every second dose months 6-12	HbA1c, We do not recommend routine complication screening in the first years after the development of ICI- induced DM, taking into account age and prognosis.	Glycemia every ICI dose during active treatment and after treatment for 6 months FUP	Glycaemia every 4-6 weeks during ICI therapy, repeat HbA1C if suspected T1DM
	Pituitary toxicity	Electolytes TSH, fT4, cortisol every 4-6 weeks	Not available	Not available	Not available	If hypophysitis is suspected: 8am cortisol, ACTH, TSH, fT4 and electrolytes; ACTH test for borderline cases; LH and testosterone levels in men and FSH and estrogen levels inpremenopausal women who experience asthenia, reduced libido, and mood swings	If any symptoms appear: 9 am cortisol (or random if unwell and treatment cannot be delayed), ACTH, TSH or FT4, LH, FSH, estradiol if premenopausal, testosterone in men, IGF-1, prolactin
	Gonadal toxicity	Not available	Not available	Testosterone every ICI dose month 1-6 every second dose months 6-12	Not available	Not available	Not available

**Table 2:** Guidelines from ESE regarding the onset and monitoring of endocrine toxicities during therapy with TKIs

**Baseline**	Thyroid toxicity	TSH, fT4
	Adrenal toxicity	ACTH, cortisol
	Mellitus diabetes	Glycemia and HbA1c
	Pituitary toxicity	Not available
	Gonadal toxicity	testosterone/estradiol
**Follow-up**	Thyroid toxicities	TSH, fT4 every 3–4 weeks at 1-6 months of TKI treatment every 2–3 months after 6 months of TKI treatment every 1 month if TKI is orally administred
	Adrenal toxicity	Periodically
	Mellitus diabetes	Periodically
	Pituitary toxicity	Data not available
	Gonadal toxicity	Data not available

Thyroid dysfunctions are the most frequent endocrine toxicities caused by ICIs and TKIs combination therapy. A biphasic pattern is frequently observed during ICIs therapy, with an initial thyrotoxic phase, followed by hypothyroidism. The diagnosis of hypothyroidism should be performed based on elevated TSH and low fT4 in primary hypothyroidism, or low fT4 with inappropriately low or normal TSH in central hypothyroidism [19]. Conversely, primary hypothyroidism or transient thyrotoxicosis due to destructive thyroiditis or a reduction in iodine uptake may result after the administration of TKIs [19].

Hypophysitis can occur more rarely. This condition is observed more frequently with anti-CTLA-4 agents, potentially resulting in multiple pituitary hormone deficiencies, while isolated ACTH deficiency has been observed, particularly in conjunction with anti–PD-1/PD-L1 agents. Magnetic resonance imaging (MRI) of the pituitary gland may reveal some suggestive diagnostic signs, such as enlargement, thickening of the stalk, or contrast enhancement, even if imaging may not always reveal abnormalities [19].

The management of primary hypothyroidism involves the administration of weight-based levothyroxine replacement, with adjustments made according to TSH levels [20]. Central hypothyroidism requires the administration of levothyroxine guided by fT4 levels, irrespective of TSH levels. Adrenal insufficiency requires prompt glucocorticoid replacement, typically with hydrocortisone in physiologic doses (15–25 mg/day), or stress-dose steroids in cases of adrenal crisis. In the case of central adrenal insufficiency, mineralocorticoid replacement therapy is not required [21]. In contrast to other immune-related AEs, most endocrine toxicities are irreversible and typically do not recover. The administration of high-dose corticosteroids is reserved for cases of hypophysitis with mass effect, substantial inflammation, or severe symptoms [22]. Typically, endocrine-AEs do not require the discontinuation of oncological treatment, if hormone replacement is adequately managed. Hormone replacement therapy is usually long-term, as endocrine toxicities may manifest late or persist for years after the discontinuation of ICIs and TKIs [23]. Coordinating efforts between the oncology and endocrinology teams is crucial for ensuring optimal patient care. The education of patients on the early recognition of symptoms, such as fatigue, hypotension, hyponatremia, and nausea, may facilitate timely diagnosis and improve outcomes of patients [24].

### Clinical trials with TKI plus ICI combined treatment and endocrine side effects

The clinical trials available in the literature regarding the use of ICIs and TKIs in combination are summarized in Table 3.

**Table 3:** Clinical trials with ICIs and TKIs in combination

AUTHORS	TRIAL	DRUGS	CANCER	Nop	GTD	HypoT	Hpit	PAI	DM
Thomas Powles et al (2020)	KEYNOTE426	Pembrolizumab + Axitinib	Advanced ccRCC	860	48%	35.4%	0.8%	0.6%	1%
Robert J. Motzer et al (2020)	JAVELIN RENAL 101	Avelumab + Axitinib	Advanced ccRCC	442	32%	24.9%	1.3%	0.4%	0.3%
Robert J. Motzer et al [34]	CLEAR	Pembrolizumab + Lenvatinib	Advanced ccRCC	356	56.8%	26%	2%	2%	0.6%
Toni K Choueiri et al (2022)	CheckMate 9ER	Nivolumab + Cabozantinib	Advanced ccRCC	323	45%	36%	2%	2%	0%
X Q Yan et al [29]	RENOTORCH	Toripalimab + Axitinib	Advanced ccRCC	210	55.8%	40.4%	0.4%	0.5%	1%
Hans J Hammers et al (2017)	CheckMate 016	Nivolumab + Ipilimumab	Advanced ccRCC	94	24.1%	13.1%	9%	0%	0%
Antonio Gonzàlez-Martìn et al (2024)	LEAP 005	Pembrolizumab + Lenvatinib	Triple-negative breast carcinoma, colon carcinoma and ovarian carcinoma	94	55%	42%	0.9%	5.1%	0.6%
Roberto Iacovelli et al [30]	TIDE-A	Avelumab + Axitinib	Advanced ccRCC	79	47.5%	23%	0.4%	0.5%	1%
W Tian et al [42]	CAP 04	Camrelizumab + Apatinib	NSCLC	58	22.2%	12.5%	5%	-	-
Shengxiang Ren et al [39]	-	Camrelizumab + Famitinib	NSCLC	41	-	12.5%	-	-	12.5%
Yuchen Zhang et al (2024)	TORAL	Toripalimab + Anlotinib	NSCLC	40	54.55%	47.5%	-	-	-
Ning et al [38]	-	TQB2450 + AL2846	NSCLC	33	-	24.2%	0.5%	-	-
Zhu X et al [43]	NCT 04984096	ICI + Anlotinib	advanced/metastatic squamous cell carcinoma of the esophagus	29	-	20.7%	-	-	-

*Nop* number of patients, *GTD* global thyroid disfunction, *HypoT* hypothyroidism, *Hpit* hypophysitis, *PAI* primary adrenal insufficiency, *DM* diabetes mellitus

#### Thyroid disfunctions

Several landmark trials have demonstrated benefits and challenges of ICI plus TKI combination therapy in cancers. Combinations of TKIs plus ICIs are the standard first-line therapy for patients with metastatic clear cell renal cell carcinoma (ccRCC), and endometrial cancer. Furthermore, these combination treatments are under investigation in many other malignancies, such as non-clear cell renal cell carcinoma (nccRCC), ovarian cancer, breast cancer, colorectal cancer (CRC), hepatocellular carcinoma (HCC), non-small cell lung cancer (NSCLC), melanoma, esophagus cancer, and thyroid cancer.

The phase III KEYNOTE-426 trial evaluated the efficacy and safety of anti-PD1 (pembrolizumab) plus a TKI (axitinib) combination therapy versus TKI (sunitinib) in monotherapy for advanced ccRCC patients. The combination arm proved an improved survival outcome with notable thyroid dysfunctions, occurring in approximately 48% of patients: hypothyroidism of any grade was found in 35.4% and hyperthyroidism in 12.8%, while severe thyroid dysfunctions (≥ grade 3) were rarely reported (hypothyroidism 0.9% and hyperthyroidism 1.2%, respectively) [25, 26]. The incidence of endocrine-AEs was lower in the TKI monotherapy arm, with 31.5% of cases of hypothyroidism and 3.8% of hyperthyroidism [25, 26]. To note, in the 43-month follow-up data, the rate of hypothyroidism rose to 36.5% [27].

In the Javelin Renal 101 trial, thyroid dysfunctions were reported as the most frequent endocrine-AEs: up to 32% of patients treated with the ICIs (avelumab) plus TKIs (axitinib) combination therapy experienced hypothyroidism [28]. In the Chinese phase III RENOTORCH study, the combination of the anti-PD1 (toripalimab) plus TKI (axitinib) in advanced RCC patients led to hypothyroidism in 40.4% of patients [29].

Similarly, the TIDE-A trial reported an overall rate of 23% of hypothyroidism [30]. Interestingly, the aim of this trial was to assess the feasibility of an intermittent strategy withdrawing the VEGFR-TKI with ICI maintenance for selected mRCC patients with evidence of response to the first-line therapy combination. The trial met the primary endpoint demonstrating that Axitinib interruption is feasible and led to a remarkably decreased rate of side effects, preserving patient quality of life. However, a significant number of patients experienced endocrine-related toxicities overall [30].

In the Nivolumab plus Cabozantinib ccRCC trial (CheckMate 9ER), hypothyroidism resulted as the third most frequent AEs, affecting over 36% of patients. Notably, severe thyroid dysfunction occurred in less than 1% of patients [25]. In the updated and subsequent publications, after 44.0 months of median survival, the most common any-grade immune-mediated AEs were hypothyroidism (27.8% patients) and hyperthyroidism (9.4%) [31, 32].

In the multicenter, open-label, parallel-cohort, dose-escalation, phase I CheckMate 016 study, hypothyroidism was reported in 33.3% of patients treated with Nivolumab plus Sunitinib, and in 20.0% of those treated with Nivolumab plus Pazopanib [33].

Lenvatinib plus Pembrolizumab was the most widely investigated combination therapy in solid tumors. The results of the CLEAR trial [34] and of LEAP 005 basket trial [35] showed that the combination therapy lenvatinib (a multitargeted TKI) and pembrolizumab (a PD-1 inhibitor) was associated with the occurrence of hypothyroidism, in approximately 42.6% of patients, and severe (grade ≥ 3) hypothyroidism in 1.1% of patients. The occurrence of hypothyroidism was attributed to lenvatinib in 56.3% of patients and to pembrolizumab in 47.2% of patients. The occurrence of thyroiditis, which had an incidence of 0.6%, was assigned to the anti-PD-1 effect, too [35]. The final analysis published with a median survival of about 4 years confirmed the efficacy and safety data of endocrine-AEs [36]. Similarly, the phase II multi-cohort LEAP-005 trial (ClinicalTrials.gov, NCT02501096) was conducted in patients with ovarian cancers and triple-negative breast cancers, reporting more frequently thyroid dysfunctions among endocrine toxicities. Another phase I trial investigated the efficacy and safety of lenvatinib and anti-PD-1 combination in unresectable intermediate-to-advanced HCC, showing a success rate of 55.4%, and an incidence of hypothyroidism of 7.1% [37]. The AL2846 trial conducted on the anti-PD-L1 and TKI combination therapy for patients with HCC showed good overall tolerability, with a hypothyroidism incidence of 24.2% [38].

Incidence of hypothyroidism was 12.5% in a phase II trial conducted in patients affected from NSCLC and treated with the combination of camrelizumab (a PD-1 inhibitor) and famitinib (an orally bioavailable receptor tyrosine kinase -RTK- inhibitor) [39]. Another study explored the efficacy and safety of toripalimab (a PD-1 inhibitor) in combination with anlotinib for the treatment of patients with advanced NSCLC, reporting hypothyroidism in 63% of the 19 enrolled patients [40]. In the CAP 04 study, hypothyroidism occurred in 22.2% of patients, and in the NSCLC cohort, hypothyroidism was reported in 12.5% [41]. Similarly, in patients treated with the combination of camrelizumab and apatinib for endometrial cancer, hypothyroidism was reported in 22.2%, subclinical hypothyroidism in 16.7%, while hyperthyroidism in a single patient (2.8%) [42].

Another study investigated the combination of anlotinib (TKI) with PD-1 inhibitors in patients with advanced or metastatic squamous cell carcinoma of the esophagus, in progression after failure of previous treatment with ICIs. All patients experienced treatment-related AEs, with thyroid dysfunction occurring at a significant rate of patients, ranging from 20.7% to 44.8% of enrolled patients [43].

#### Other rare endocrine toxicities: hypophysitis, adrenal insufficiency, diabetes and electrolytes disorders

Although thyroid dysfunctions remain the most prevalent endocrine-related toxicities associated with ICIs, other rare endocrine AEs, including adrenal insufficiency, hypophysitis, diabetes mellitus, and electrolyte disorders, have been recorded across various trials.

The phase III trial KEYNOTE-426 trial reported cases of adrenal insufficiency in approximately 0.8% of patients and of hypophysitis in about 0.6% of patients [19]. In a study investigating sintilimab (an anti-PD-1 mAb) plus anlotinib (a TKI) as first-line therapy in patients with advanced NSCLC, only one case of adrenal insufficiency was observed (4.5%) [44]. Contrariwise, in CheckMate 9ER trial, adrenal insufficiency was reported in 2% of patients included and treated with nivolumab (an anti-PD-1 mAb) plus cabozantinib (TKI) combination [25]. Data from CLEAR trial and LEAP 005 basket trial reported adrenal insufficiency in 5.1% and of hypophysitis in 0.9% of patients treated with pembrolizumab (an anti-PD-1 mAb) plus lenvatinib (RTK) in combination. Notably, these adverse events were mostly referred to an autoimmune reaction triggered by pembrolizumab (an anti-PD-1 mAb). Finally, the CLEAR trial and LEAP 005 basket trial revealed other rare endocrine-Aes, such as diabetes mellitus (0.6%) and hypocalcaemia (1.4%) [34, 35]. Hyperglycaemia was reported only in few trials, with an incidence of around 11%–12% of cases [39, 42].

### Management of endocrine toxicities related to ICI plus TKI treatment and evidence-based recommendations

Treatment strategies depend on the specific hormone dysfunction and onset time. Several key strategies are suggested for the management of endocrine toxicities related to ICI and TKI combination treatment, as reported in Fig. 1.

The integration of symptoms and signs, biochemical markers, hormone assessment may contribute to early diagnoses endocrine-AEs [9, 19, 45]. Patients should be educated on the potential onset of signs and symptoms, that may be suggestive for endocrine dysfunctions.

Periodical screening of endocrine function before and during the combination treatment is essential for early detection and treatment of endocrine dysfunctions. No guidelines and suggestions are available on the pre-treatment and follow-up screening of endocrine-AEs in patients on ICIs plus TKIs combination therapy.

A multidisciplinary approach, through collaboration between oncologists and endocrinologists, is crucial in the management of complex endocrine AEs, especially in distinguishing between immune-mediated and TKI-related side effects [9].

In addition to standard endocrine replacement therapies, modifications of the cancer treatment may be required depending on the severity of the endocrine toxicities [46]. Dose adjustments of oncological drugs, both delays and reductions, are allowed with TKIs and ICIs and are generally appropriate for a safety patient’s management [32, 47]. However, most cases of ICIs-related AEs are manageable only with hormone replacement therapies [47, 48]. During the administration of oncological treatments, it is recommended continuative endocrinological follow-up, in particular for patients who have experienced multiple or severe endocrine events [49, 50].

#### Management of thyroid dysfunctions

Thyroid dysfunctions are the most frequently encountered endocrine toxicities caused by ICIs and TKIs combination therapy [19, 51–53].

Thyroid dysfunction typically follows a biphasic pattern: thyrotoxicosis occurs on average within 2–6 weeks of starting treatment, lasting approximately 5–6 weeks, followed by the development of hypothyroidism [45, 54–57]. In most studies, post-thyroiditis hypothyroidism is persistent: many patients remain on levothyroxine therapy for over 1 year and the levothyroxine requirement is like that of an “athyroid” subject, suggesting almost complete destruction of the gland [45, 54–57].

As summarized in Table 1, thyroid functional tests (at least TSH and fT4 levels) should be evaluated before starting ICI according respectively to national and international guidelines [45, 54–57] and before starting TKIs, according to experts’ advice [58, 59]. The timing of screening during ICIs is not univocal: the monitoring of thyroid function is suggested before each ICIs dose during 3–12 months of treatment, or at least before every second ICI cycle during 3-6 months of treatment.

Thyroiditis is typically a painless condition, with mild or absent symptoms. If a rigorous surveillance protocol is applied, thyroid dysfunction is usually detected by routine blood tests [19, 23]. Usually, patients develop self-resolving thyrotoxicosis or subclinical hypothyroidism, that do not require treatment or discontinuation of combined ICI plus TKI therapy [23]. A diagnosis of overt hypothyroidism should be considered in patients who report fatigue, weight gain, cold intolerance, dry skin, or constipation [23, 51–53]. The diagnosis is confirmed by fT3 and fT4 values below the laboratory reference range, and elevated TSH level [23, 51–53]. The assessment of anti-thyroglobulin (anti-TG) and anti-thyroid peroxidase (anti-TPO) antibodies may provide further information of etiopathogenesis of thyroiditis in ICIs and TKIs therapies. In particular, the presence of pre-treatment anti-TPO and anti-TG autoantibodies may contribute to the pathogenesis of thyroid endocrine-AE. However, the assessment of anti-thyroid antibodies does not change management and is not routinely required.

Concomitant low TSH, fT3 and fT4 levels may suggest central hypothyroidism, which requires further investigations to rule out hypophysitis, hypopituitarism, pituitary metastases, or empty sella syndrome secondary to metastatic spread of cancer to the brain [23]. True hyperthyroidism during treatment with ICI + TKI combination was never reported [25–27, 31, 51–53]; however, if hyperthyroidism is persistent, the evaluation of anti-thyroid stimulating hormone receptor antibodies and thyroid ultrasound to exclude Graves’ disease is recommended. In patients with primary thyroid dysfunction, the thyroid ultrasound scan is recommended to evaluate the presence of thyroid nodules and/or metastatic spread of thyroid gland [19, 23].

The treatment of thyrotoxicosis requires the use of beta-blockers in patients with tachycardia; in addition, temporary suspension of therapy with ICIs or TKIs is necessary until the symptoms and signs related to thyrotoxicosis resolve. The use of thyrostatic drugs should only be considered in cases of thyrotoxicosis with severe symptoms, due to the high risk of developing severe hypothyroidism [25–27, 31].

In patients with primary hypothyroidism, levothyroxine replacement therapy should always be started at a low dose and then should be adjusted as required. The levothyroxine assessment should be approximately 1.6 mcg/kg/day in individuals under the age of 60 years and without comorbidities [23]. Otherwise, in old individuals and in patients with undated onset of hypothyroidism, it is advisable to start with lower levothyroxine dose (25 or at most 50 mcg/day), to re-evaluate TSH and thyroid hormones every 4–6 weeks for a correct dose titration [19, 23].

Before starting hormone replacement therapy with levothyroxine in hypothyroidism, it is suggested to evaluate plasma ACTH and serum cortisol levels to rule out concomitant adrenal insufficiency [23]. In the latter case, it is advisable to correct firstly the adrenal insufficiency and then the hypothyroidism, to avoid the risk of an adrenal crisis [19, 23, 51–53].

In our clinical practice, monitoring of TSH and thyroid hormones is required every 2–4 weeks for the first 12 weeks, and subsequently immediately before each cycle of ICI+TKI. Once the drugs are discontinued, thyroid function continues to be monitored on a clinical basis [19, 23].

#### Management of hypophysitis

Given the potentially lethal consequences of hypopituitarism (mainly ACTH deficit) resulting from hypophysitis, the early diagnosis of this condition is crucial because it requires a multidisciplinary and timely approach [23, 53, 60, 61].

ICIs, particularly anti-CTLA-4 and, less frequently, anti-PD-1/PD-L1, can induce isolated ACTH deficiency, hypopituitarism and hypophysitis [62]. Hypophysitis is diagnosed in patients with active pituitary inflammation (identified as pituitary enlargement on MRI) with or without headache, or with combination of headache and new onset hypopituitarism; ICI-induced hypopituitarism is diagnosed in patients with deficiency of two or more pituitary axes, with or without evidence of clinical or radiological hypophysitis; isolated ACTH deficiency is diagnosed in patients morning cortisol below assay specific reference range with non-elevated ACTH, in the absence of exogenous glucocorticoids and in absence of other pituitary hormone disfunctions [62]. A stimulation cosyntropin test (a synthetic analogue of ACTH) may be useful in confirming the diagnosis of secondary adrenal insufficiency and should be considered for patients with diagnosis in doubt, as those with chronic use of glucocorticoids [23]. In common clinical practice, it remains essential to carefully evaluate diagnostic timing to correct adrenal insufficiency, as soon as possible, avoiding therapeutic delays that could be fatal for the patient.

TKIs can contribute with direct effects on the secretion of hypothalamic-pituitary hormones and on glandular perfusion [23, 53, 60, 61].

Diagnosis may be delayed due to the nonspecific nature of the symptoms, if ACTH and cortisol are not routinely performed during the monitoring of ICIs and TKI complications [53, 60, 61]. Pituitary dysfunction may occur weeks or months after the start of therapy [23, 53, 60, 61]. Therefore, according to international society guidelines (as summarized in Table 1), at least glucose, electrolytes, TSH, fT4, cortisol levels should be tested before starting ICIs, although a comprehensive pituitary hormone evaluation is suggested from the British and French society of Endocrinology and from European Society on Endocrinology, in patients considered at high risk for pituitary toxicity, such as those on treatment with anti-CTLA-4. Moreover, when symptoms attributable to ACTH deficit or to hypopituitarism occur (such as general malaise, loss of appetite, asthenia, nausea, hypotension or headache, general malaise, loss of appetite), it is mandatory to perform a complete pituitary hormonal evaluation (ACTH, cortisol, TSH, fT4, electrolytes and plasma osmolarity, LH, FSH, prolactin, testosterone/oestradiol, chemical/physical examination of urine).

In patients with biochemical diagnosis of hypopituitarism or with new onset headache, a contrast-enhanced MRI scan of the pituitary gland should be performed to reveal signs of inflammation of the pituitary gland, and to exclude focal lesion or cancer metastatic dissemination to pituitary region or brain [19, 23].

Hormonal replacement therapy for ascertained central adrenal insufficiency should be promptly prescribed with parenteral or oral administration of corticosteroid (hydrocortisone or acetate cortisone), according to patients’ clinical condition. Moreover, as mentioned above, before starting levothyroxine replacement therapy, also in patients with central hypothyroidism, it is essential to rule out adrenal corticosteroid deficiency and start corticosteroid replacement therapy (if clinically indicated), to avoid acute adrenal crisis [19, 23].

Sex hormone replacement therapy should be considered in both men and women, if clinically appropriate [23]. However, testosterone therapy is contraindicated in several conditions, such as increased haematocrit, thrombophilia, untreated obstructive sleep apnea, uncontrolled heart failure, recent myocardial infarction or stroke (within six months), severe urinary disorders, as well as in the presence of breast or prostate cancer [23]. GH replacement therapy is typically contraindicated in cancer patients with active disease and during active surveillance [19].

Cancer treatment may be temporarily withdrawn, since symptoms of acute ACTH deficit and/or hypopituitarism recover, and may be generally resumed with caution, after the improvement and resolution of signs and symptoms related to pituitary disfunctions [23]. There are no indications to definitive withdrawal of cancer therapy in patients who developed ICIs/TKIs related pituitary dysfunction. A multidisciplinary assessment with oncologists and endocrinologists should be performed to evaluate the withdrawal of ICIs and/or TKIs, that should be reserved to selected patients with severe neurological involvement (headache, visual field defects, or cranial nerve palsy) [23].

Long-term endocrinological follow-up remains essential, as hormonal deficiencies, particularly ACTH deficiency is typically permanent, while thyroid or gonadal deficiencies may partially recover [19, 23].

#### Management of primary adrenal insufficiency

Primary adrenal insufficiency may develop with acute, subacute, chronic onset. The symptoms reflect the time of development of hormone impairment and the patient’s concomitant clinical conditions [19, 53, 63–65]. Nausea, anorexia, arterial hypotension, abdominal pain, weight loss, and hypoglycaemia are the most typical symptoms. According to international society guidelines for endocrine toxicity in ICIs treatment and according to expert suggestions for endocrine toxicity in TKIs (as summarized in Table 2), electrolytes and serum cortisol should be evaluated before the first treatment dose. ACTH assessment should be limited to patients with cortisol levels < 500 nmol/L. Suggestions for surveillance of primary adrenal insufficiency was provided only by the French Endocrine Society, that proposed the assessment of serum sodium and cortisol before each ICI dose during first 6 months of treatment and every second cycle in the following 6 months.

Diagnostic work-up in patients with low levels of serum cortisol should include also the assessment of adrenocorticotrophic hormone (ACTH) and anti-21 hydroxylase antibodies (optional; not usually positive). Cosyntropin test may be performed in patients with suspected diagnosis of PAI, according to clinical patient’s condition, without postponing the start of glucocorticoid replacement therapy.

Treatment with 15-30 mg hydrocortisone per oral administration should be administered in patients with asymptomatic or mild PAI. Treatment with 30–100 mg hydrocortisone should be administered in patients with moderate/severe symptoms of PAI, through oral or parenteral administration according to clinical condition of patient [63, 64]. Stress dose (i.e. 50–100 mg i.v. hydrocortisone four times daily) should be considered for sick patients with Addison crisis, acute surgery, severe infection, sepsis. The assessment of corticosteroid replacement therapy should be tailored based on the patient’s clinical condition. Monitoring of glucocorticoid replacement therapy should rely mainly on clinical evaluation, including assessment of body weight, blood pressure, signs suggestive of glucocorticoid excess/deficiency and biochemical assessment, mainly electrolytes assessment [19, 63–65].

Prednisone and dexamethasone are not preferred to hydrocortisone and cortisone acetate for the higher risk of Cushingoid side-effects and suppression of the hypothalamic–pituitary–adrenal axis (HPA) [66, 67].

Patients should be educated on the use of stress‑dose of glucocorticoids and on the parenteral administration of hydrocortisone in specific conditions, such as infections, sepsis, or hemodynamic instability. Prompt self‑administration by the patient or a trained caregiver can help prevent or reduce the severity of adrenal crises [19, 63–65].

For those with primary adrenal insufficiency, it is important to consider whether mineralocorticoid replacement with fludrocortisone is needed. This is particularly relevant in cases of persistent low sodium, high potassium, or proven aldosterone deficiency, as mineralocorticoid therapy can help maintain electrolyte balance and prevent orthostatic hypotension [19, 63–65].

#### Screening, diagnosis and management of type 1 mellitus diabetes

As suggested by the AIOM guideline, glycemia should be monitored before first dose and before each dose during active ICIs/TKIs treatment, and every month after ICI discontinuation for 6 months of follow-up. In patients with suspected diabetes mellitus, laboratory test should include the assessment of plasmatic electrolytes, kidney function, urinary and blood ketones, anion GAP, bicarbonates, anti-pancreatic insula (ICA) and anti-glutamic acid decarboxylase (GAD) antibodies, and C-peptide. Results suggestive of a type 1 diabetes mellitus diagnosis are hyperglycaemia with a low C-peptide or positive autoantibodies.

The management of type 1 diabetes developing during combination therapy with ICIs and TKIs requires an urgent, proactive and personalized approach [19, 53]. Given that insulin deficiency in these patients is often severe and irreversible, insulin therapy is generally the first-line treatment, as in type 1 diabetes [19]. The treatment aim is to normalize fasting and postprandial blood glucose levels, preventing the acute and chronic complications associated with diabetes [19, 53]. Basal-bolus regimens are typically started to provide stable basal insulin coverage along with rapid-acting insulin to control postprandial glucose levels. The initial dose should be carefully determined based on body weight and glucose measurements, with careful adjustment to achieve optimal control while avoiding hypoglycaemia [19, 53]. In selected cases with highly variable glucose levels, continuous subcutaneous insulin infusion may be appropriate [53].

As in type 1 diabetes, oral hypoglycaemic drugs are not indicated in ICI-related mellitus diabetes, that is mediated by the autoimmune/autoinflammatory destruction of beta cells caused by immune-mediated mechanisms [23]. In parallel, TKI-related hyperglycaemia and mellitus diabetes seem to be mediated more from insulin resistance than from absolute insulin deficiency, as proved by plasma C-peptide levels. Therefore, in patients with mellitus diabetes occurring during TKIs, treatment with metformin may be considered if renal function is preserved; while other agents such as dipeptidyl peptidase 4 (DPP-4) inhibitors or glucagon-like peptide 1 (GLP-1) receptor agonists may be considered [19, 23, 53]. According to pleiotropic effect, GLP-1 analogues may be prescribed in patients with residual pancreatic, to benefit also from the reduction of cardiovascular events, improvement of dyslipidaemia and weight loss [53]. The use of sodium-glucose cotransporter 2 (SGLT2) inhibitors should be considered with extreme caution due to the risk of euglycemic diabetic ketoacidosis, particularly in this subgroup of patients [19, 23, 53].

Follow-up care must be structured and continuous. Frequent self-monitoring of blood glucose levels is essential, possibly using a sensor, especially during the early stages of treatment and insulin titration, but also subsequently in chronic management [53].

Patients should be taught to recognize the symptoms of both hypoglycaemia and hyperglycaemia and should receive instructions on how to adjust their insulin doses when necessary [19, 23].

Laboratory monitoring should include regular measurements of fasting blood glucose, glycated hemoglobin, and C-peptide, if appropriate, at a frequency appropriate to the stability of glycaemic control and the stage of cancer treatment [19, 23, 53].

Coordination between endocrinology and oncology teams is essential to ensure that metabolic control does not interfere with cancer therapy and to adjust treatment plans according to changes in clinical status [19, 23, 53].

In the long term, patients should undergo periodic reassessment of insulin requirements, screening for microvascular and macrovascular complications, and education training to support treatment adherence and quality of life [19, 23].

## Conclusions

The combination therapy involving ICI and TKI is associated with a higher incidence of endocrine complications, particularly thyroid dysfunction and disorders of carbohydrate metabolism. The identification of etiopathogenetic mechanisms of endocrine toxicities mediated by ICIs and TKIs is useful for correct management of endocrine and cancer treatments. Therefore, multidisciplinary management is required to early diagnose endocrine toxicities, safely treat the patients and guarantee adherence to ICI and TKI therapies.

## Data Availability

No datasets were generated or analysed during the current study.
